# Supplementary material for: Spinal Solitary Fibrous Tumors: An Original Multicenter Series and Systematic Review of Presentation, Management, and Prognosis
Source: Cancers (Basel). 2022 Jun 8;14(12):2839. doi: 10.3390/cancers14122839 (PMC9221085; doi:10.3390/cancers14122839)
Supplement: Supplementary file 1 [file cancers-14-02839-s001.zip › Spinal SFT - Sup figure S1.pdf]

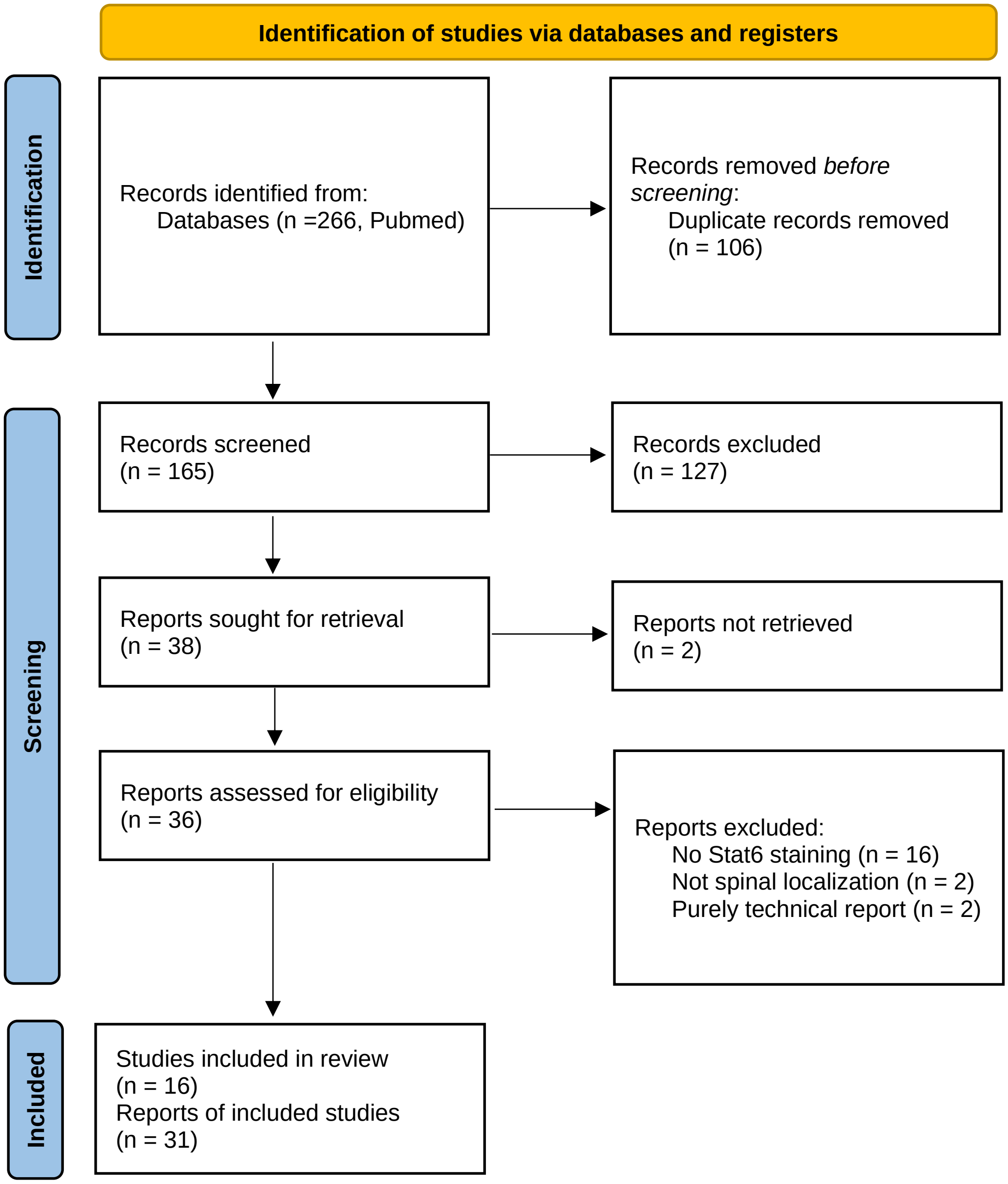

Sup. Figure S1: Flow-chart of the literature review for systematic review according to the PRSIMA guidelines (adapted from Page et al, 2021).

Sup. Table S1: Detailed anonymous data retrieval from our series and the systematic literature review for Stat6 positive spinal SFT/HPC (csv file).
